# Adaptive EWMA control chart for monitoring the coefficient of variation under ranked set sampling schemes

**DOI:** 10.1038/s41598-023-45070-x

**Published:** 2023-10-17

**Authors:** Afshan Riaz, Muhammad Noor-ul-Amin, Walid Emam, Yusra Tashkandy, Uzma Yasmeen, Javed Rahimi

**Affiliations:** 1 COMSATS University Islamabad-Lahore Campus, Lahore, Pakistan; 2 Department of Statistics and Operations Research, King Saud University, Riyadh, Saudi Arabia; 3 Department of Statistics, Brock University, Saint Catharines, Canada; 4 Horticulture Department, Kabul City Agriculture and Food Processing Institute, Kabul, Afghanistan

**Keywords:** Computational science, Statistics

## Abstract

In this study, we introduce an Adaptive Exponentially Weighted Moving based Coefficient of Variation (AEWMCV) control chart, designed to address situations where the process mean fluctuates over time and the standard deviation of the process changes linearly with the process mean. To enhance the efficiency and effectiveness of the control chart, we integrate the ranked set sampling method and its modified schemes, such as Simple Random Sampling, Quartile RSS, Median RSS, and Extreme RSS. The performance of the proposed AEWMCV control chart and the studied CV control charts are evaluated using the Average Run Length and Standard Deviation of Run Length metrics. Our findings reveal that the proposed control chart outperforms the existing CV control charts, especially in detecting slight to moderate changes in the process CV. To illustrate the practical applicability of the suggested control chart, we present an example demonstrating its use on a real dataset. The results highlight the superior performance of the AEWMCV control chart in accurately detecting and responding to changes in the process CV. In conclusion, our study introduces an innovative AEWMCV control chart that combines ranked set sampling and its modified schemes to enhance performance in scenarios with fluctuating process means and changing standard deviations. The proposed control chart proves to be more effective in detecting subtle variations in the process CV compared to traditional CV control charts. This research provides a valuable contribution to the field of control chart methodology, especially when dealing with challenging or costly data collection scenarios.

## Introduction

Statistical Process Control (SPC) aims to achieve two primary objectives: first, it seeks to detect deviations in performance from the ideal state, and second, it aims to determine the underlying causes of unfavorable variations. A widely used tool in various industries to visualize and monitor data over time is the control chart. Control charts for variables can be designed using a time-weighted approach to effectively identify minor shifts in the process. By utilizing time-weighted techniques, control charts can efficiently capture subtle changes and fluctuations in the process. These charts offer a graphical representation of the monitored data, allowing businesses to observe trends and patterns over time. By promptly detecting and addressing minor drifts in the process, organizations can maintain quality standards, reduce waste, and optimize their operations. Overall, statistical process control and the use of time-weighted control charts play crucial roles in ensuring consistent product or service quality and facilitating continuous improvement within businesses.

The memory-type control charts combine current data with information about previous samples. Temporal weighting in SPC can be effectively demonstrated through the EWMA control chart, originally introduced by^1^. This method employs a weighted average of past data points to monitor the stability and detect shifts in a process over time. The EWMA control chart assigns more significance to recent data points while gradually reducing the impact of older data as they move further into the past. This characteristic allows the chart to be more responsive to subtle process changes and provides a real-time assessment of process performance. The EWMA chart is more sensitive to tiny and moderate alterations than the conventional Shewhart type control chart. In certain processes, the issue of inertia necessitates simultaneous control of both large and tiny shifts. Inertia refers to the phenomenon where a process may exhibit resistance to immediate changes, leading to delays in response to small or large shifts in the process. Abbas et al.^2^ introduced progressive mean control charts. Abbas et al.^3^ utilized progressive setup for developing the EWMA control chart.

To effectively manage the inertia issue, it becomes essential to implement control mechanisms that are capable of detecting and addressing both significant and subtle variations in the process. By doing so, the process can be kept under close observation, enabling prompt corrective actions when needed, regardless of the magnitude of the shifts. It makes use of an adaptive weighted control chart technique to deal with the issues. The memory-type adaptive control charts have recently drawn a lot of interest due to their exceptional all-around performance at various shift sizes. As effective control charts that are utilized to improve the detection capabilities in the online monitoring process, numerous charts based on the adaptive EWMA (AEWMA) and adaptive CUSUM (ACUSUM) have been described in the literature. For instance, the AEWMA mean control chart developed with the Huber function (^4^) by^5^ aided in the creation of a suitable admixture of Shewhart and EWMA control charts. A CUSUM control chart with a two-dimensional Markov chain model was introduced by^6^. The effectiveness of the AEWMA control chart in signaling linear drifts was examined by^7^. According to^8^ the performance of an AEWMA control chart based on estimated parameters is more negatively impacted than it is in the case of known values. Aly et al.^9^ demonstrated that the ideal design parameters may be evaluated for any desired in-control *ARLs* by using the optimal design parameter to analyze the wide range of mean shifts using a probability distribution. By^10^, an effective AEWMA mean control chart is suggested. Aly et al.^11^ developed and designed the AEWMA control chart for zero-inflated passion process. Mitra et al.^12^ presented the AEWMA control chart where the degree of shift in process mean varing over a wide range of values. Nazir et al.^13^ focused on developing a robust AEWMA control chart and explored its applications in the manufacturing process. Noor‐ul‐Amin and Noor^14^ presented the AEWMA control chart for monitoring the process mean in Bayesian theory with different loss functions. Recently^15–18^, presented adaptive-based memory control charts and proved that the adaptive-based control charts have better performance than traditional EWMA control charts.

In SPC, one fundamental rule is that it has constant mean and variance in an in-controlled process that is normally distributed. The chart is said to OOC in the presence of the shift in mean and/or variance. The online monitoring through the traditional $\overline{X},$ *S* and *R* charts fails when the process mean is considered as in-control (IC) but fluctuates it time to time and SD is a linear change with the mean of the process. In such cases, the CV is used in control charts. The CV is construed as a risk for investors in the finance field where it is associated with the volatility of the return that is equal to the expected value of the return on an asset^19^. For the validation of quality work in the field of biological and chemical science, the CV plays an important role^20^. Kang et al.^21^ summarized that the utilization of CV control charts in clinical chemistry is instrumental in addressing control problems and maintaining the required quality control checks. Regularly repeated measurements of critical characteristics enable healthcare professionals to proactively identify and address potential issues, thereby upholding the quality and accuracy of clinical test results. Hong et al.^22^ reported that CV based EWMA (EWMCV) control chart has efficient performance with the smallest OOC $ARLs$. Castagliola et al.^23^ introduced one-sided two EWMCV charts for unknown shift sizes based on the squared CV and implemented the suggested chart on a dataset taken from the manufacturing of zinc alloy parts. Castagliola et al.^24^ used three parametric logarithmic transformations to make the CV chart more efficient than the considered CV charts. They reported that by using optimal chart parameters the suggested control chart was superior to the considered CV control charts. The application of the suggested control chart on a dataset gathered from the manufacturing of zinc alloy parts. Khaw et al.^25^ presented CV control charts for variable sample size (VSS) and for sampling interval (SI) based where the average time to signal and expected time to signal were used as a performance measuring tool. In recent times, CV control chart based on EWMA statistic under the RSS scheme was presented by^26^. Lee et al.^27^ presented double sampling CV control chart. Tran and Heuchenne^28^ presented CV control chart by using the variable sampling based CUSUM control chart. Arshad et al.^29^ suggested a function based CV control chart for monitoring the shifts in CV.

From the literature, it is observed that the performance of control charts can be enhanced through the RSS scheme and its modified forms. An efficient and useful sampling design named RSS was introduced by^30^. Takahasi and Wakimoto^31^ mathematically demonstrated how the sample mean of RSS serves as an unbiased estimator of the population mean, exhibiting the desirable property of statistical accuracy. Additionally, this estimator possesses a smaller variance compared to other traditional sampling methods. Numerous researchers utilized RSS in control charts such as^32–38^ and^39^.

Over the past decade, attention has been drawn towards the AEWMA control charts due to their exceptional performance in detecting small to moderate shifts in the process. Throughout the literature, considerable emphasis has been placed on the effectiveness of AEWMA control charts in various applications. Surprisingly, the AEWMCV control chart, which combines the adaptive exponentially weighted moving average approach with the coefficient of Variation (CV) measure, has not been previously discussed or explored in the existing literature. This novel integration presents a promising avenue for addressing quality control challenges in situations where the process mean fluctuates over time and the standard deviation of the process is linked to the process mean. The lack of prior discussion on the AEWMCV control chart makes it a potential breakthrough and a promising direction for further research and application in diverse industries and processes. In this research study, we introduce a novel control chart called the AEWMCV chart. The chart is designed to detect process mean shifts of unknown magnitude using an EWMA statistic at the initial stage. Based on this estimator, an appropriate smoothing constant value is selected to construct the AEWMA plotting statistic. The selection of the smoothing constants is based on the size of the detected shift; smaller smoothing constants are chosen for smaller shifts, and vice versa. The proposed AEWMCV control chart is developed within the framework of RSS-based schemes. To provide a comprehensive understanding of RSS and its modified schemes, Sect. "Ranked set sampling" presents detailed mathematical formulations and setups. In Sect. "Proposed control chart", we present the mathematical development of the suggested AEWMCV control chart, explaining its construction and implementation procedures. To assess the performance of the proposed control chart, simulation results are presented and discussed in Sect. "Performance evaluation". Additionally, we conduct a comparative study between the suggested AEWMCV control chart and the conventional AEWMCV control chart considered in this study, which is presented in Sect. "Performance comparison". In Sect. "Real data application", we demonstrate the practical application of the suggested control chart by applying it to a real dataset collected from the manufacturing process of zinc alloy parts.

## Ranked set sampling

Ranked set sampling is a non-random sampling technique that is particularly useful when the cost of acquiring information about the population elements is high. It is often employed in situations where obtaining precise measurements for the entire population is expensive or time-consuming. In RSS, the population is first divided into ordered sets based on certain characteristics of interest. The ranks are then randomly selected, and data is collected only from the elements within the chosen ranks. This sampling method ensures that information gathered from low and extreme characteristics is measured, thereby improving efficiency compared to simple random sampling. The elementary knowledge of RSS schemes and the modified schemes such as classical RSS, extreme RSS, and median RSS is presented in this section.

### Classical ranked set sampling

The efficient sampling design named RSS was introduced by^30^. Numerous authors utilized RSS for estimation purposes instead of SRS. For the selection of a ranked-set sample, a large number of units are randomly selected from the interested population without enumerating the data relative to the study variable. Randomly assigned these units into different specific equal size sets and then on the basis of auxiliary information or expert judgment, ranked the units of each set. The demonstration of the RSS procedure is given as:

(i) Pick $k^{2}$ units at random from the under study population, where *k* stands for set size.
(ii) Randomly distribute $k^{2}$ units into m groupings of *k* units each.
(iii) The *k* units of each set are visually grouped using an auxiliary variable or by any other method that is free of charge, without identifying the precise measurements of the data about the research variable.
(iv) The lowest-ranked unit from the initial set is quantified after ranking all *k* sets. Similarly, the second-lowest ranked unit from the second set is quantified, and so on, until the largest ranked unit from the set of *k* units is quantified.
(v) The entire process comprising steps (i-iv) is iteratively repeated 'r' times to obtain the desired sample size. This repetitive procedure is carried out to ensure that an adequate amount of data is collected, meeting the required sample size criteria. i.e. $n = kr$.

Takahasi and Wakimoto^31^ proved the properties of RSS *i.e.*

1 $$ \overline{Y}_{RSS} = \frac{1}{n}\sum\limits_{i = 1}^{k} {Y_{i(i:k)} } ,\;\;\;\;{\text{where}} E(\overline{Y}_{RSS} ) = \mu_{y} , $$

2 $$ {\text{and}} \sigma_{RSS}^{2} = Var(\overline{Y}_{RSS} ) = \frac{{\sigma_{y}^{2} }}{n} - \frac{1}{{n^{2} }}\sum\limits_{i = 1}^{k} {(\mu_{y(i:k)} - \mu_{y} } ). $$

it is assumed that the sets and quantified units are to be independent in RSS. In RSS, the emphasis is placed on selecting the most representative sample by prioritizing the true measurements of highly representative units. Additionally, the method incorporates supplementary knowledge about the target population to enhance the sampling process. This combination of using relevant measurements and additional population insights ensures a more accurate and reliable representation of the larger population in the sample selection.

### Median ranked set sampling

Another efficient and modified sampling design within the RSS framework for estimating the population mean is known as the MRSS scheme. This method is an enhancement of the conventional RSS approach and is designed to improve the accuracy and robustness of population mean estimation introduced by^40^. The step-by-step plan of MRSS is demonstrated as:

(i) Randomly select *k* units from the given population, where *k* represents the set size.
(ii) Randomly distribute these units into *k* (equal size)sets.
(iii) For even sample size m, choose the (*m*/2)th ranked unit from the first set and the (*m*/2)th ranked unit from the last set.
(iv) Select the (*k* + 1)/2 ranked units from all sets for odd sample size *k*.

These steps outline the process of randomly choosing units, dividing them into sets, and determining which ranked units to select based on whether the sample size is even or odd.

It constitutes a full cycle of r independent samples, each of size k. The entire procedure can be repeated r times to conduct multiple cycles of independent sampling, with each cycle consisting of k units selected randomly.

For even and odd cases, with MRSS the sample mean and variance are given respectively,

3 $$ \overline{Y}_{O,MRSS} = \frac{1}{k}\sum\limits_{i = 1}^{k} {X_{i((k + 1)/2:k))} } ,\;\;\;\overline{Y}_{E,MRSS} = \frac{1}{k}\left( {\sum\limits_{i = 1}^{k/2} {X_{i(k/2:k)} } + \sum\limits_{i = 1}^{k} {X_{i((k + 1)/2:k))} } } \right). $$

4 $$ \sigma_{MRSS}^{2} = Var(\overline{Y}_{O,MRSS)} ) = \frac{1}{k}\sigma_{Y(k + 1/2:k)}^{2} ,\;\;\;\sigma_{MRSS}^{2} = Var(\overline{Y}_{E,MRSS)} ) = \frac{1}{2k}\left( {\sigma_{Y(k/2:k)}^{2} + \sigma_{Y(k + 2/2:k)}^{2} } \right). $$

### Extreme ranked set sampling

The modified scheme of the traditional RSS in the estimation procedure of the population mean is called ERSS, as introduced by^30^. The steps involved in the ERSS as follows:

(i) Randomly select *k* units from the given population, where *k* represents the set size.
(ii) Distribute the k units randomly into k sets, each with a size of *k.*
(iii) For an even sample size, select the first ranked unit from the first (*k*/2) sets and the last ranked unit from the last (*k*/2) sets.
(iv) For an odd sample size, select the first ranked unit from the first (*k* + 1)/2 sets, the last ranked unit from the next (*k−*1)/2 sets, and the middle unit from the last set.

To achieve the desired sample size of *n* = *2rk*, repeat the entire procedure r times. The selection of the lowest/largest units from each set can be done visually or by using any other easily applicable method. This modified ERSS procedure aims to estimate the population mean and provides an alternative approach to the traditional RSS method^41^. The selection of ERSS samples is as follows:

Let $$X_{i1} ,X_{i2} ,........X_{ik}$$ be a random sample with size *k* then $$\left\{ {X_{1(1)} ,X_{1(k)} ,X_{2(1)} X_{2(k)} ,....X_{k(1)} ,X_{k(k)} } \right\}$$ is an ERSS with size *2 k* where $$i = 1,2...k.$$$$X_{i(k)} = \max \left\{ {X_{i1} ,X_{i2} ,........X_{ik} } \right\},$$ and $$X_{i(1)} = \min \left\{ {X_{i1} ,X_{i2} ,........X_{ik} } \right\}.$$ The properties such as mean and variance of ERSS is given respectively,

5 $$ \overline{Y}_{ERSS,E} = \frac{1}{k}\left[ {\sum\limits_{i = 1}^{k/2} {Y_{i(1)} + \sum\limits_{i = 1}^{k/2} {Y_{{\frac{k}{2} + i(k)}} } } } \right],\;\;\overline{Y}_{ERSS,O} = \frac{1}{k}\left[ {\sum\limits_{i = 1}^{(k - 1)/2} {Y_{i(1)} } + \sum\limits_{i = 1}^{(k - 1)/2} {Y_{{\frac{(k - 1)}{2} + i(1)}} + Y_{{k(\frac{k + 1}{2})}} } } \right] $$

6 $$ \sigma_{ERSS,E}^{2} = \frac{1}{2k}\left[ {\sigma_{(1)}^{2} + \sigma_{k}^{2} } \right],\;\;\sigma_{ERSS,O}^{2} = \frac{k - 1}{{2k^{2} }}\left[ {\sigma_{(1)}^{2} + \sigma_{(k)}^{2} } \right] + \frac{1}{{k^{2} }}\left[ {\sigma_{{\frac{(k + 1)}{2}}}^{2} } \right] $$

### Quartile ranked set sampling

To reduce ranking errors, especially in cases with small sample sizes, Takahasi and Wakimoto^31^ introduced the quartile RSS (QRSS) as a method that yields an efficient mean estimator. The demonstration of the ERSS procedure is as follows:

(i) Randomly select k units from the given population, where *k* represents the set size.
(ii) Randomly distribute the k units into k sets, each with a size of *k*, and then rank them according to the variable of interest.
(iii) For an even sample size, select the q1(*k* + 1)th ranked unit from the first k/2 samples, and from the next *k*/2 samples, select the *q*_3_(*k* + 1)th ranked unit. Here, *q*_*1*_ is the first quartile (0.25) and *q*_*3*_ is the third quartile (0.75).
(iv) When the sample size is odd, for actual measurements, select the q1(*k* + 1)th unit from the first (*k*-1)/2 samples, and from the further (*k*-1)/2 samples, select the *q*_3_(*k* + 1)th unit. Additionally, choose the median unit from the last sample data for the QRSS. To obtain a sample of size rn, repeat the entire process *r* times.

The QRSS method improves the estimation of the population mean by considering quartiles and offers advantages in situations where small sample sizes can lead to ranking inaccuracies. This approach helps achieve a more efficient estimation process for the mean. The population mean estimator using QRSS with *r*=1 is for an even sample size,

7 $$ \overline{Y}_{QRSS,E} = \frac{1}{k}\left[ {\sum\limits_{i = 1}^{k/2} {Y_{{i(\frac{k + 1}{4})}} } + \sum\limits_{i = 1}^{k} {Y_{{\frac{k}{2} + i\left( {\frac{3(k + 1}{4}} \right)}} } } \right], $$

with variance

8 $$ \sigma_{QRSS,E}^{2} = \frac{1}{2k}\left[ {\sigma_{{\left( {\frac{k + 1}{4}} \right)}}^{2} + \sigma_{{\left( {\frac{3(k + 1)}{4}} \right)}}^{2} } \right], $$

and for odd sample size

9 $$ \overline{Y}_{QRSS,O} = \frac{1}{k}\left[ {\sum\limits_{i = 1}^{(k - 1)/2} {Y_{{i\left( {\frac{k + 1}{4}} \right)}} } + \sum\limits_{i = 1}^{(k - 1)/2} {Y_{{\frac{(k - 1)}{2} + i\left( {\frac{3(k - 1}{4}} \right)}} + Y_{{k\left( {\frac{k + 1}{2}} \right)}} } } \right] $$

with variance

10 $$ \sigma_{QRSS,O}^{2} = \frac{k - 1}{{2k^{2} }}\left[ {\sigma_{{\left( {\frac{k + 1}{4}} \right)}}^{2} + \sigma_{{\left( {\frac{3(k + 1)}{4}} \right)}}^{2} } \right] + \frac{1}{{k^{2} }}\left[ {\sigma_{{\left( {\frac{k + 1}{2}} \right)}}^{2} } \right] $$

## Proposed control chart

For the precise results, Kang^21^ presented the CV control chart with repeated measurements of some characteristics. In the last decade, the CV control charts got the attention of the researchers and they introduced Shewhart and EWMA type CV control charts. To check the online monitoring of the Shewhart CV type control chart, Castagliola et al.^24^ recommended a statistic that follows the normal distribution with zero mean and unit variance. The statistic is given by

11 $$ T_{t} = a + b\ln (\widehat{\gamma }_{t} - c), $$

here $$\widehat{\gamma }_{t} = {\raise0.7ex\hbox{${\sigma_{t} }$} \!\mathord{\left/ {\vphantom {{\sigma_{t} } \mu }}\right.\kern-0pt} \!\lower0.7ex\hbox{$\mu $}}_{t}$$ is defined the CV at a time $$t.$$ The parameters ($$a,b,c$$) in CV control chart belong to the log-normal distribution and it is right-skewed distribution. The following formulas are given below to evaluate the parameters

12 $$ b = \frac{{F_{N}^{ - 1} (r)}}{{\ln \left( {\frac{{y_{0.5} - y_{r} }}{{y_{1 - r} - y_{0.5} }}} \right)}}, $$

13 $$ a = - b\ln \left( {\frac{{y_{0.5} - y_{r} }}{{1 - \exp \left( {\frac{{F_{N}^{ - 1} (r)}}{b}} \right)}}} \right), $$

14 $$ c = y_{0.5} - e^{{ - \frac{a}{b}}} , $$

The $a, \, b$ and *c* are constants that are specified according to sample size and the value of CV such that $a,b > 0$. For more details see^26^.

Muttlak and Al-Sabah^32^ introduced the AEWMA control chart for monitoring the process mean. In the first stage, they supposed an estimator with an unknown process mean shift (δ) using the EWMA statistic. Based on this estimator, an appropriate value of "λ" is selected, which is used in the AEWMA plotting statistic. The classical EWMA statistic with sample mean $\overline{Y}_{t}$ is given by.

In summary, the AEWMA control chart employs an adaptive approach to estimate the process mean shift and select a suitable value of "λ" leading to improved monitoring of the process mean over time.

15 $$ E_{t} = \lambda \overline{Y}_{t} + (1 - \lambda )E_{t - 1} ,\;\;\;E_{0} = \mu ,\;\;0 < \lambda \le {1} $$

where $\lambda$ is a smoothing constant. When $\lambda$ = 1, the EWMA control chart approaches to Shewhart control chart and the EWMA control chart can detect small to moderate shifts when $\lambda$ = 0. Haq et al.^10^ suggested the unbiased estimator of $E_{t}$ is defined as follows

16 $$ \widetilde{\delta }_{t}^{**} = \frac{{E_{t} }}{{1 - (1 - \lambda )^{t} }} $$

For the IC process $$E(\widetilde{\delta }_{t}^{**} ) = 0$$ for time *t*. The $$\delta$$ is not known in advance, we consider $$\widetilde{\delta }_{t} = \left| {\widetilde{\delta }_{t}^{**} } \right|$$ $$\theta (\widetilde{\delta }_{t} ) \in (0,1]$$ is defined as

$$ \theta (\widetilde{\delta }_{t} ) = \left\{ \begin{gathered} 0.015{\text{ if 0}}{.00 < }\widetilde{\delta }_{t} \le 0.25 \hfill \\ 0.10{\text{ if 0}}{.25 < }\widetilde{\delta }_{t} \le 0.75 \hfill \\ 0.20{\text{ if 0}}{.75 < }\widetilde{\delta }_{t} \le 1.00 \hfill \\ 0.25{\text{ if 1}}{.00 < }\widetilde{{\delta_{t} }} \le 1.50 \hfill \\ 0.50{\text{ if 1}}{.50 < }\widetilde{\delta }_{t} \le 2.50 \hfill \\ 0.80{\text{ if 2}}{.50 < }\widetilde{\delta }_{t} \le 3.50 \, \hfill \\ {1}{\text{.00 if 3}}{.50 < }\widetilde{\delta }_{t} \hfill \\ \end{gathered} \right\} $$

To detect negative and positive shifts in the process the AEWMA plotting statistic is defined as

17 $$ E_{t}^{*} = \theta (\widetilde{{\delta_{t} }})Y_{t} + (1 - \theta (\widetilde{{\delta_{t} }}))E_{t - 1}^{*} $$

In the current study, we introduced AEWMCV-SRS, AEWMCV-RSS, AEWMCV-ERSS, AEWMCV-MRSS, and AEWMCV-QRSS mean control chart for the phase-II process monitoring. The CV ($\widehat{\gamma }$) of a random variable $Y$ with mean $\mu_{y}$ and SD $\sigma_{y}$ is given by

18 $$ \hat{\gamma } = \frac{{\sigma_{y} }}{{\mu_{y} }}, $$

by definition, $\widehat{\gamma }$ is defined $\left( { - \infty , + \infty } \right)$. Let $Y_{1} ,Y_{2} ,Y_{3} ....,Y_{k}$ be a simple random sample of size $k$ with respective sample mean and *SD* are symbolized as $\overline{Y}_{t,SRS}$ and $S_{t,SRS}$, the CV in case of SRS for time $t$ is given by

19 $$ \widehat{\gamma }_{t,SRS} = \frac{{S_{t,SRS} }}{{\overline{Y}_{t,SRS} }},\;\;S_{t,SRS} = \sqrt {\frac{1}{m - 1}\sum\limits_{t = 1}^{m} {(Y_{t} - \overline{Y})^{2} } } . $$

The IC and OOC CV are denoted with $\widehat{\gamma }_{0}$ and $\widehat{\gamma }_{1}$ respectively. Where $\tau = \frac{{\widehat{\gamma }_{1} }}{{\widehat{\gamma }_{0} }}\%$ denotes the shift size in the CV. The EWMCV plotting statistic under SRS after using the three parametric logarithmic transformations is given by

20 $$ E_{t,SRS} = \lambda T_{t,SRS} + (1 - \lambda )E_{t - 1,SRS} ,\;E_{0,SRS} = \mu_{SRS} = 0, $$

the respective unbiased estimators of $E_{(SRS)t}$ is defined as

21 $$ \widetilde{\delta }_{(SRS)t}^{**} = \frac{{E_{(SRS)t} }}{{1 - (1 - \lambda )^{t} }}, $$

The AEWMCV plotting statistics *¯*based on SRS and RSS are defined as:

$$ E_{(SRS)t}^{*} = \theta (\widetilde{\delta }_{t} )Y_{(SRS)t} + (1 - \theta (\widetilde{\delta }_{t} ))E_{(SRS)t - 1}^{*} , $$

For the RSS and its modified schemes, Let $Y_{1(1,k)} ,Y_{2(2,k)} ,...,Y_{k(k,k)}$ be a sample of size $k$ in case of RSS that are selected with the following method described in Sect. "Ranked set sampling". Here we consider. The CV ($\hat{\gamma }$) based EWMCV-RSS test statistic at time *t* is defined in (22) under RSS is given by

22 $$ E_{(RSS),t} = \lambda T_{(RSS),t} + (1 - \lambda )E_{(RSS),1 - t} ,\;\;E_{(RSS),0} = \mu_{(RSS)} = 0, $$

where $$\widehat{\gamma }_{(RSS),t} = \frac{{S_{(RSS),t} }}{{\overline{Y}_{(RSS),t} }},$$ $$S_{(RSS),t} = \sqrt {{\text{var}} (\overline{Y}_{RSS} )}$$

The AEWMCV-RSS statistic $^{ - } E_{(RSS)t}^{*}$ (plotting statistic) using $Y_{(RSS)t}$ is given by

23 $$ E_{(RSS)t}^{*} = \theta (\widetilde{{\delta_{t} }})Y_{(RSS)t} + (1 - \theta (\widetilde{{\delta_{t} }}))E_{(RSS)t - 1}^{*} ,\;\;E_{(RSS)0}^{*} = 0. $$

24 $$ {\text{and}} \widetilde{\delta }_{(RSS),t}^{**} = \frac{{E_{(RSS),t} }}{{1 - (1 - \lambda )^{t} }}. $$

The AEWMCV-ERSS statistic $^{ - } E_{(R)t}^{*}$ (plotting statistic) using $Y_{(R)t}$ is given by

25 $$ E_{(ERSS)t}^{*} = \theta (\widetilde{{\delta_{t} }})Y_{(ERSS)t} + (1 - \theta (\widetilde{{\delta_{t} }}))E_{(ERSS)t - 1}^{*} ,\;\;E_{(ERSS)0}^{*} = 0. $$

$$ {\text{and}} \widetilde{\delta }_{(ERSS),t}^{**} = \frac{{E_{(ERSS),t} }}{{1 - (1 - \lambda )^{t} }}. $$

The AEWMCV-MRSS statistic $^{ - } E_{(MRSS)t}^{*}$ (plotting statistic) using $Y_{(MRSS)t}$ is given by

26 $$ E_{(MRSS)t}^{*} = \theta (\widetilde{{\delta_{t} }})Y_{(MRSS)t} + (1 - \theta (\widetilde{{\delta_{t} }}))E_{(MRSS)t - 1}^{*} ,\;\;E_{(MRSS)0}^{*} = 0. $$

and $$\widetilde{\delta }_{(MRSS),t}^{**} = \frac{{E_{(MRSS),t} }}{{1 - (1 - \lambda )^{t} }}.$$

The AEWMCV-QRSS statistic $^{ - } E_{(QRSS)t}^{*}$ (plotting statistic) using $Y_{(QRSS)t}$ is given by

27 $$ E_{(QRSS)t}^{*} = \theta (\widetilde{{\delta_{t} }})Y_{(QRSS)t} + (1 - \theta (\widetilde{{\delta_{t} }}))E_{(QRSS)t - 1}^{*} ,\;E_{(QRSS)0}^{*} = 0. $$

and $$\widetilde{\delta }_{(QRSS),t}^{**} = \frac{{E_{(QRSS),t} }}{{1 - (1 - \lambda )^{t} }}.$$

The control limit is denoted with *h* and can be calculated desired fixed in-control *ARL*. If plotting statistics $E_{(RSS),t}^{*} ,E_{(ERSS)t}^{*} ,E_{(MRSS)t}^{*} , \, E_{(QRSS)t}^{*} ,E_{(SRS)t}^{*}$ located outside the threshold values $h_{SRS}$ and $h_{RSS,ERSS,MRSS,QRSS}$, the control charts indicate the process is OOC.

## Performance evaluation

We have studied the AEWMCV control chart with RSS and its modified schemes by taking sample sizes i.e., $n = 3$ and 5 with different values of $\lambda$. To calculate $ARL$ and $SDRL$ numerous approaches are available in the literature, Monte-Carlo simulations is one of them. In the current study, the run length properties are evaluated through the Monte-Carlo simulation process. Table 1 presented the transformed statistic parametric values $a,b,c$ values of design schemes for making them normal with respect to $\widehat{\gamma }$, and $n$, where the statistic $T_{t} = a + b\ln (\widehat{\gamma }_{t} - c),$ follows the standard normal distribution. By following the^42,43^, and ^37^, It is assumed that for an IC state of the process a large dataset is available and the control limit *h* of suggested charts is estimated using perfect ranking by using these samples. The constant value of $h/L$(where *L* is a parameter for EWMA statistic) for the suggested chart specified for desired *ARLs.*

**Table 1:** Parametric values for sampling schemes.

Control charts	$\hat{\gamma }$	*a*		*b*		*c*		*h/L*	
		*n* = 3	*n* = 5	*n* = 3	*n* = 5	*n* = 3	*n* = 5	*n* = 3	*n* = 5
EWMACV-SRS	0.25	3.1610	4.1021	3.1699	3.9999	− 0.1633	− 0.1326	4.66	5.99
	0.15	5.3010	6.9996	4.2699	5.5112	− 0.1633	− 0.1426	4.73	6.16
	0.05	14.2273	16.7	8.9999	8.8789	− 0.1633	− 0.1078	4.65	6.19
AEWMCV-SRS	0.25	3.1266	4.1244	3.1027	3.9987	− 0.1576	− 0.1296	0.6976	0.7086
	0.15	5.4124	6.8801	4.1112	5.4112	− 0.1426	− 0.1426	0.7477	0.7105
	0.05	14.2451	18.3374	8.8627	10.9987	− 0.1576	− 0.1426	0.7132	0.6972
AEWMCV-RSS	0.25	3.2401	4.7452	3.2112	4.6898	− 0.1301	− 0.1139	0.7562	0.7309
	0.15	5.4114	7.7312	4.3112	6.3312	− 0.1426	− 0.1426	0.7663	0.7163
	0.05	13.7564	23.3099	8.3187	15.1029	− 0.1426	− 0.1626	0.7149	0.7853
AEWMCV-ERSS	0.25	3.2401	4.5131	3.2112	4.0112	− 0.1301	− 0.0116	0.7562	0.8158
	0.15	5.4114	7.3613	4.3112	6.7112	− 0.1426	− 0.1426	0.7663	0.7273
	0.05	13.7564	20.2099	8.3187	13.6005	− 0.1426	− 0.1626	0.7149	0.7021
AEWMCV-MRSS	0.25	4.8384	7.4116	3.8112	5.1248	− 0.1426	− 0.1139	0.7318	0.7076
	0.15	7.4634	12.2124	5.0112	7.9982	− 0.1426	− 0.1426	0.7436	0.7212
	0.05	18.8940	33.5099	10.0112	19.9999	− 0.1226	− 0.1626	0.7112	0.7049
AEWMCV-QRSS	0.25	3.2401	6.0112	3.2112	5.1819	− 0.1301	− 0.1426	0.7562	0.7139
	0.15	5.4114	9.5364	4.3112	6.8112	− 0.1426	− 0.1426	0.7663	0.7096
	0.05	13.7564	27.4410	8.3187	16.9005	− 0.1426	− 0.1626	0.7149	0.7313

To estimate the OOC *ARL* and *SDRL* values of the suggested AEWMCV control chart we used simulated samples from Phase-II with a shifted process. Tables 2 and 3, presented the results of the AEWMCV chart with different values of smoothing constant(λ) i.e., 0.05, 0.15 and 0.25 for specified $ARL_{0} = 370$ by using sample sizes such as 3 and 5. The following findings are acquired from the results presented in Tables 2 and 3.

(i) From Tables 2 and 3, we observed that from small to moderate shifts in the CV, the suggested AEWMCV- SRS, RSS, QRSS, MRSS, and ERSS control chart quickly detected an OOC signal. For example, in Table 2 at 5% shift with $n = 5$, the $ARLs$ are 176.00, 151.14, 121.15, 137.05, 134.60, and 143.77 with EWMCV-SRS, AEWMCV-SRS, AEWMCV-RSS, AEWMCV-ERSS, AEWMCV-MRSS, and AEWMCV-QRSS respectively. It is revealed that the AEWMCV-RSS control chart is superior to the EWMCV-SRS control chart as quickly detects the OOC situation by reducing *ARLs*.
(ii) It is inferred from results in Tables 2 and 3 that early detection of the OOC process due to the smallest value of $\hat{\gamma }.$ For example, with shift 5% at $n = 5$, the $ARLs$ of AEWMCV-SRS in Table 2 are 110.57, 126.74, and 151.14 at $\hat{\gamma } = 0.05,0.15$ and 0.25 respectively. Further in Table 3 at the same parameters the $ARLs$ are 133.35, 157.81, and 194.02 with $\hat{\gamma } = 0.05,0.15$ and 0.25, it can be observed that the suggested charts are efficiently performed with a smaller value of $\hat{\gamma },$ the similar results are obtained as ^44^.

**Table 2:** *ARLs* (*SDRLs*) of suggested control chart with *n* = 5.

Shift (%)	$\hat{\gamma }$	EWMCV-SRS	AEWMCV-SRS	AEWMCV-RSS	AEWMCV-ERSS	AEWMCV-MRSS	AEWMCV-QRSS
0	0.25	375.90 (380.31)	376.49 (464.25)	374.22 (464.74)	367.50 (458.11)	366.48 (450.91)	374.50 (474.13)
	0.15	371.61 (375.25)	374.67 (465.31)	368.84 ( 451.04)	365.65 (460.57)	376.60 (475.70)	370.81 (463.04)
	0.05	370.14 (367.12)	376.34 (461.30)	368.79 (477.24)	370.53 (441.16)	369.94 (440.22)	371.34 (463.92)
5	0.25	176.00 (174.75)	151.14 (183.04)	121.15 (146.78)	137.05 (219.53)	134.60 (161.57)	143.77 (177.57)
	0.15	174.25 (172.57)	126.74 (153.89)	103.68 (124.46)	92.22 (111.34)	124.04 (150.45)	123.28 (150.55)
	0.05	116.11 (109.19)	110.57 (131.02)	95.05 (120.40)	79.78 (91.63)	95.66 (108.19)	94.01 (112.93)
10	0.25	72.69 (68.67)	57.16 (66.69)	41.29 (49.31)	46.61 (58.96)	50.43 (57.54)	50.95 (60.44)
	0.15	70.61 (66.80)	48.70 (56.97)	36.77 (42.71)	29.67 (34.84)	46.76 (55.29)	45.79 (52.53)
	0.05	50.786 (45.57)	44.00 (50.29)	32.45 (40.45)	27.11 (30.82)	40.89 (45.38)	37.13 (43.99)
20	0.25	23.96 (20.07)	17.48 (20.45)	11.59 (13.37)	10.28 (11.91)	15.99 (18.80)	14.92 (17.34)
	0.15	22.72 (19.05)	15.24 (18.01)	10.63 (12.39)	7.98 (8.89)	14.63 (17.62)	13.26 (15.70)
	0.05	18.733 (15.08)	14.12 (16.44)	9.11 (10.99)	7.42 (8.37)	13.28 (15.49)	11.33 (13.51)
30	0.25	12.55 (9.61)	8.41 (9.66)	5.62 (6.05)	4.78 (4.73)	7.70 (8.65)	6.96 (7.91)
	0.15	11.80 (9.16)	7.42 (8.47)	5.23 (5.48)	4.05 (4.07)	7.11 (8.22)	6.42 (7.23)
	0.05	10.128 (7.66)	6.88 (7.79)	4.49 (4.71)	3.77 (3.72)	6.63 (7.39)	5.56 (6.29)
50	0.25	6.00 (4.32)	3.69 (3.67)	2.61 (2.29)	2.31 (1.94)	3.43 (3.40)	3.13 (3.04)
	0.15	5.52 (3.99)	3.35 (3.28)	2.44 (2.14)	2.10 (1.59)	3.20 (3.12)	2.92 (2.76)
	0.05	5.017 (3.59)	3.14 (3.01)	2.23 (1.91)	1.14 (1.48)	3.13 (2.98)	2.68 (2.48)
100	0.25	2.55 (1.64)	1.71 (1.25)	1.34 (0.76)	1.23 (0.60)	1.59 (1.07)	1.49 (0.97)
	0.15	2.35 (1.50)	1.59 (1.09)	1.30 (0.71)	1.17 (0.50)	1.55 (1.06)	1.46 (0.94)
	0.05	2.16 (1.34)	1.52 (1.00)	1.24 (0.62)	1.14 (0.43)	1.51 (0.99)	1.40 (0.48)
200	0.25	1.42 (0.76)	1.15 (0.41)	1.05 (0.30)	1.03 (0.21)	1.12 (0.40)	1.10 (0.37)
	0.15	1.34 (0.63)	1.12 (0.45)	1.04 (0.29)	1.01 (0.20 m)	1.11 (0.46)	1.08 (034)
	0.05	1.270 (0.54)	1.11 (0.48)	1.01 (0.19)	1.01 (0.20)	1.11 (0.57)	1.09 (0.48)

**Table 3:** *ARLs* (*SDRLs*) of suggested control chart with *n* = 3.

Shift (%)	$\hat{\gamma }$	EWMCV-SRS	AEWMCV-SRS	AEWMCV-RSS	AEWMCV-ERSS	AEWMCV-MRSS	AEWMCV-QRSS
0	0.25	370.78 (374.40)	369.41 (458.04)	370.40 (476.67)	370.40 (476.67)	370.30 (476.34)	370.40 (476.67)
	0.15	374.38 (377.58)	368.71 (476.97)	365.43 (464.75)	365.43 (464.75)	371.49 (465.18)	365.43 (464.75)
	0.05	369.83 (373.93)	368.42 (450.48)	373.54 (462.21)	373.54 (462.21)	368.82 (455.73)	373.54 (462.21)
5	0.25	204.29 (204.73)	194.02 (225.82)	191.83 (254.57)	191.83 (254.57)	166.86 (210.84)	191.83 (254.57)
	0.15	194.97 (193.30)	157.81 (210.52)	147.60 (191.15)	147.60 (191.15)	143.32 (177.34)	147.60 (191.15)
	0.05	170.69 (170.38)	133.35 (159.53)	135.15 (164.75)	135.15 (164.75)	140.68 (172.62)	135.15 (164.75)
10	0.25	105.60 (102.19)	83.78 (100.63)	81.78 (104.85)	81.78 (104.85)	74.70 (91.64)	81.78 (104.85)
	0.15	98.71 (95.97)	72.65 (90.61)	62.60 (79.18)	62.60 (79.18)	68.04 (79.71)	62.60 (79.18)
	0.05	84.92 (83.16)	62.53 (73.74)	57.73 (69.31)	57.73 (69.31)	65.13 (78.52)	57.73 (69.31)
20	0.25	39.61 (35.89)	29.52 (34.69)	25.61 (32.34)	25.61 (32.34)	26.12 (31.64)	25.61 (32.34)
	0.15	37.01 (33.51)	25.37 (31.70)	20.54 (25.81)	20.54 (25.81)	25.82 (28.90)	20.54 (25.81)
	0.05	32.58 (29.89)	23.12 (27.44)	19.86 (23.94)	19.86 (23.94)	23.20 (27.84)	19.86 (23.94)
30	0.25	21.11 (17.89)	15.03 (17.91)	12.23 (15.33)	12.23 (15.33)	13.46 (16.64)	12.23 (15.33)
	0.15	19.87 (16.87)	12.90 (16.13)	10.29 (12.83)	10.29 (12.83)	12.33 (15.22)	10.29 (12.83)
	0.05	17.68 (15.35)	12.03 (14.47)	9.98 (12.15)	9.98 (12.15)	12.49 (14.96)	9.98 (12.15)
50	0.25	9.91 (7.84)	6.46 (7.45)	5.31 (6.06)	5.31 (6.06)	5.81 (6.71)	5.31 (6.06)
	0.15	9.26 (7.40)	5.62 (6.45)	4.60 (5.16)	4.60 (5.16)	5.45 (6.21)	4.60 (5.16)
	0.05	8.26 (6.78)	5.34 (6.07)	4.42 (4.88)	4.42 (4.88)	5.34 (6.06)	4.42 (4.88)
100	0.25	4.08 (3.02)	2.60 (2.37)	2.20 (1.97)	2.20 (1.97)	2.45 (2.29)	2.20 (1.97)
	0.15	3.75 (2.80)	2.36 (2.17)	1.99 (1.69)	1.99 (1.69)	2.32 (2.09)	1.99 (1.69)
	0.05	3.38 (2.58)	2.27 (2.01)	1.98 (1.67)	1.98 (1.67)	2.28 (2.05)	1.98 (1.67)
200	0.25	2 (1.85)	1.63 (1.02)	1.35 (0.82)	1.35 (0.82)	1.44 (0.91)	1.35 (0.82)
	0.15	1.92 (1.26)	1.43 (0.94)	1.30 (0.72)	1.30 (0.72)	1.43 (0.89)	1.30 (0.72)
	0.05	1.74 (1.09)	1.40 (0.87)	1.27 (0.68)	1.27 (0.68)	1.39 (0.84)	1.27 (0.68)

It is declared from Tables 2 and 3 results, that the overall performance of the suggested AEWMCV control charts are superior to their competitors with minimum $ARLs$ and $SDRLs$.

## Performance comparison

The comparison section is made between suggested and considering the CV control charts in terms of $ARLs$ and $SDRLs.$ At a certain shift, a control chart is said to be superior and has minimum OOC *ARLs*. Table 4 presents the comparison between the suggested chart with considered EWMCV control charts with the specified parameters such as $\lambda = 0.1$,$n = 5$ and $\widehat{\gamma } = 0.05$ at $ARL_{0} = 370.$ In Table 4, the $ARLs$ are 159.43, 177.25, 116.11, and 108.59 for EWMCV, DEWMCV, SEWMCV, and REWMCV control charts at $\tau = 5\%$ respectively while the *ARLs* with suggested charts are 110.57, 95.05, 79.78, 95.66, and 94.04 with AEWMCV-SRS, RSS, ERSS, MRSS, and QRSS respectively. It is inferred that through suggested control charts under RSS schemes, the early detection of the OOC situation causes an increase in shift size. Based on $ARL$ values, it is inferred that the RSS-based AEWMCV control chart is superior to considered CV control charts.

**Table 4:** Comparison between the suggested control chart and considered charts based on *ARLs.*

Shift (%)	$\hat{\gamma }$		EWMCV	DEWMCV	SEWMCV	REWMCV	AEWMCV				
							SRS	RSS	ERSS	MRSS	QRSS
5	0.05	*ARLs*	159.43	177.25	116.11	108.59	110.57	95.05	79.78	95.66	94.01
		*SDRLs*	163.78	183.49	109.19	103.17	131.02	120.40	91.63	108.19	112.93
10	0.05	*ARLs*	64.87	64.47	50.786	42.19	44.00	32.45	27.11	40.89	37.13
		*SDRLs*	63.33	63.07	45.57	37.69	50.29	40.45	30.82	45.38	43.99
20	0.05	*ARLs*	20.06	19.946	18.733	14.42	14.12	9.11	7.42	13.28	11.33
		*SDRLs*	17.60	17.46	15.08	11.22	16.44	10.99	8.37	15.49	13.51
30	0.05	*ARLS*	10.21	10.395	10.128	7.81	6.88	4.49	3.77	6.63	5.56
		*SDRLs*	8.34	8.93	7.66	5.72	7.79	4.71	3.72	7.39	6.29
50	0.05	*ARLS*	4.721	4.705	5.017	3.77	3.14	2.23	1.14	3.13	2.68
		*SDRLs*	3.75	4.04	3.59	2.55	3.01	1.91	1.48	2.98	2.48
100	0.05	*ARLs*	1.993	1.918	2.169	1.70	1.52	1.24	1.14	1.51	1.40
		*SDRLs*	1.28	1.40	1.34	0.94)	1.00	0.62	0.43	0.99	0.48
200	0.05	*ARLs*	1.218	1.17	1.270	1.11	1.11	1.01	1.01	1.11	1.09
		*SDRLs*	0.50	0.48	0.54	0.34	0.48	0.19	0.20	0.57	0.48

## Real data application

**Figure 1:**
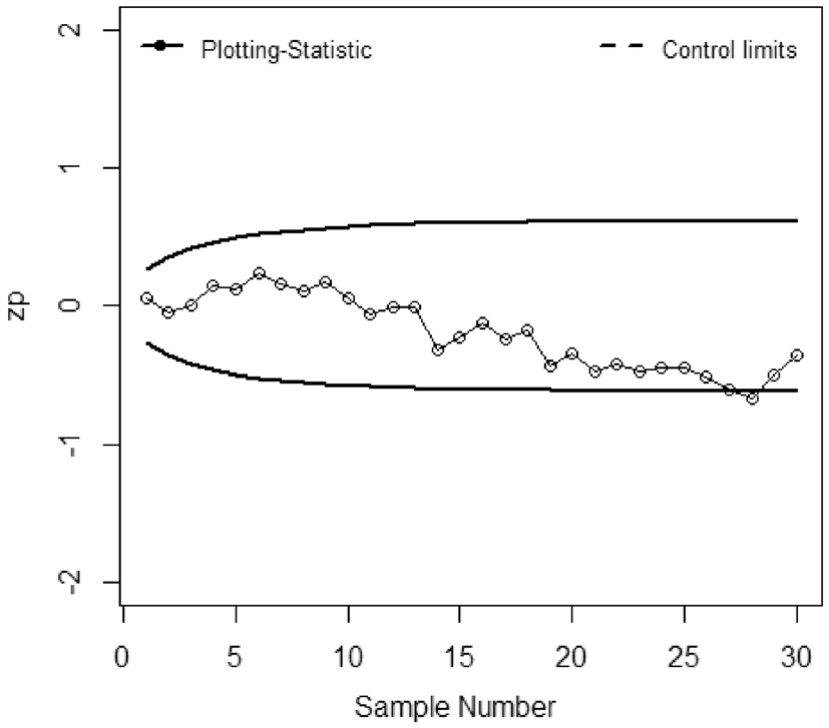
EWMCV-RSS control chart.

**Figure 2:**
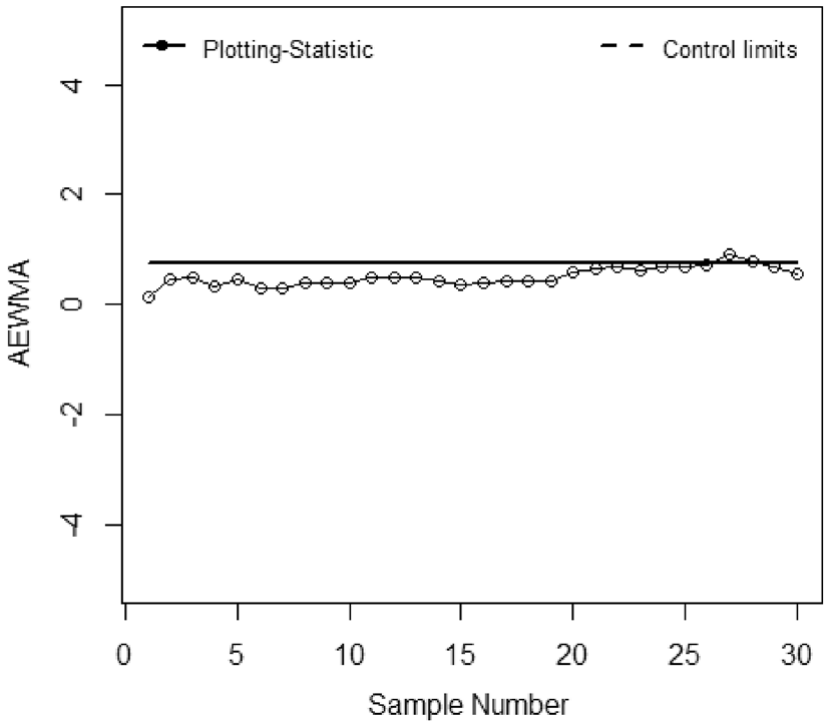
AEWMCV-SRS control chart.

**Figure 3:**
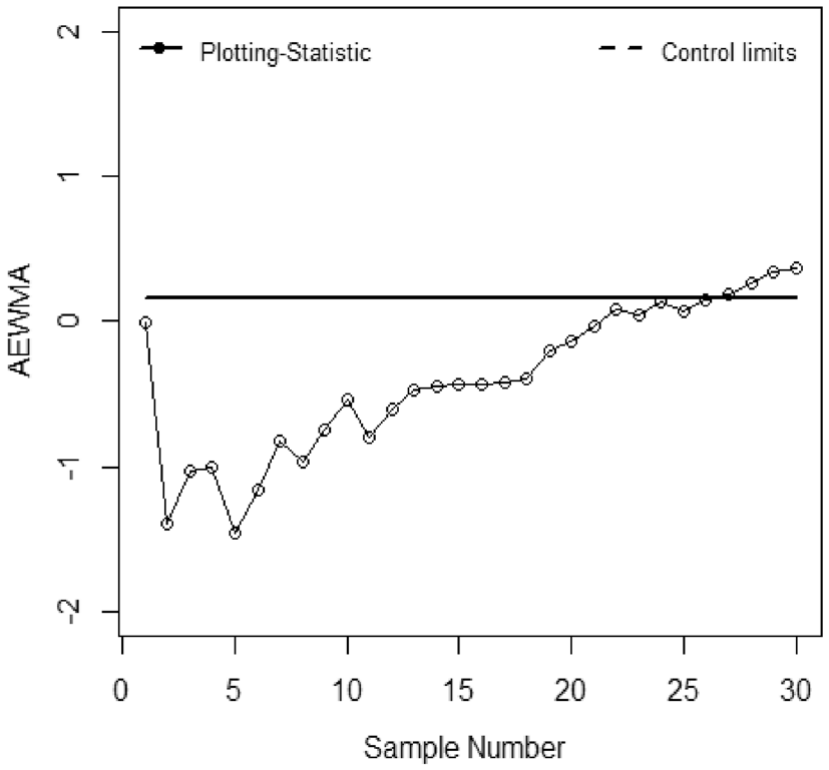
AEWMCV-RSS control chart.

**Figure 4:**
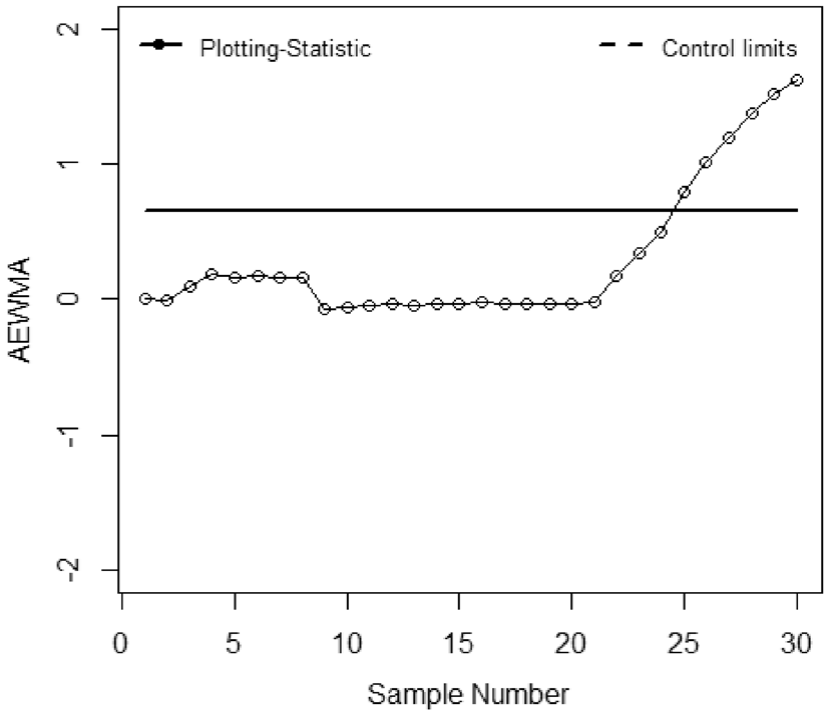
AEWMCV-ERSS control chart.

**Figure 5:**
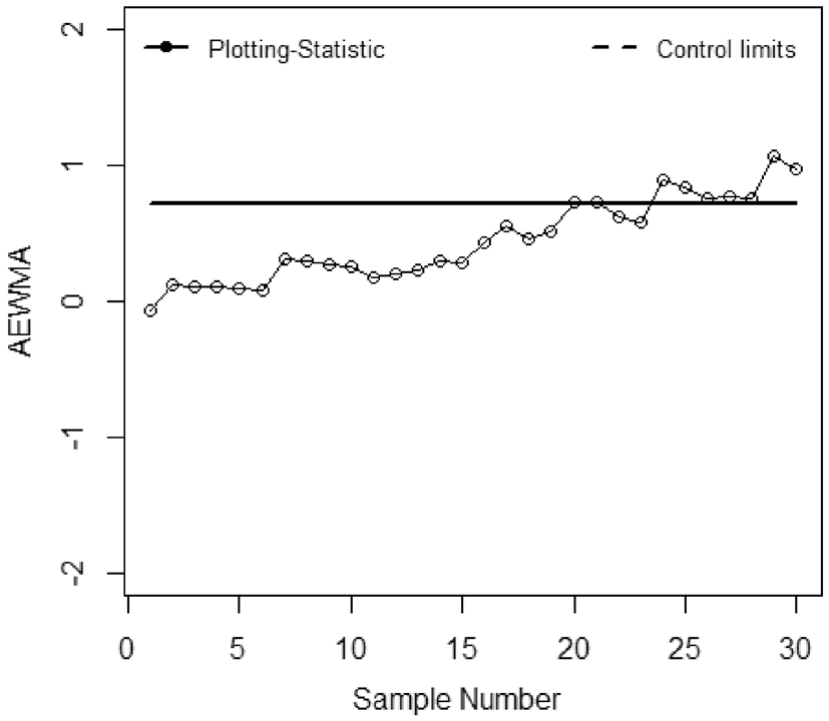
AEWMCV-MRSS control chart.

**Figure 6:**
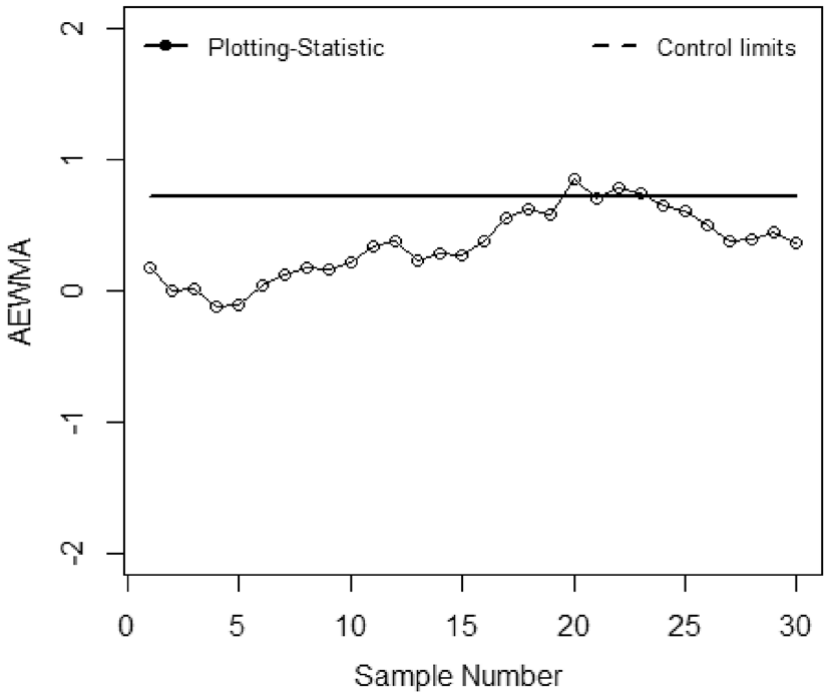
AEWMCV-QRSS control chart.

The application of the suggested chart on the manufacturing process of zinc alloy parts for the sanitary sector is given in^24^. The weight (*Y*) (measured in grams) of scrap zinc material on $n$ identical part codes (codes can change during inspection). The parameters *a, b,* and *c* are computed from the phase I dataset under SRS and RSS each with sample size 5 by considering the process as IC we have computed with $\widehat{\gamma } = 0.01$ for *ARL*_0_ = 200. Table 5 presented the phase II dataset with 30 samples of size 5, plotting statistics (*E*_*t*_), $h_{SRS}$ and $h_{R}$ under SRS and RSS schemes. The first 20 observations are selected from the IC process and the remaining 10 observations are from the shifted process i.e., $\tau$ = 2. Figures 1, 2, 3, 4, 5 and 6 present the out-of-control points in a real data application. We have compared the performance of EWMCV-RSS control chart with AEWMCV by using SRS, RSS, ERSS, and MRSS.

**Table 5:** The respective control limits and statistics of the suggested and EWMCV-RSS control charts.

S. no.	AEWMCV-SRS		AEWMCV-RSs		AEWMCV-ERSS		AEWMCV-MRSS		AEWMCV-QRSS		EWMCV-RSS	
	$E_{SRS}^{*}$	$h_{SRS}$	$E_{R}^{*}$	$h_{R}$	$E_{R}^{*}$	$h_{R}$	$E_{R}^{*}$	$h_{R}$	$E_{R}^{*}$	$h_{R}$	$E_{t}$	$LCL$
1	0.1545	0.7496	− 0.0022	0.1604	− 0.0012	0.6463	− 0.0696	0.7163	0.1738	0.7107	0.0555	− 0.2674
2	0.4712	0.7496	− 1.3899	0.1604	− 0.0024	0.6463	0.1206	0.7163	0.0040	0.7107	− 0.0424	− 0.3597
3	0.5062	0.7496	− 1.0240	0.1604	0.1012	0.6463	0.1094	0.7163	0.0153	0.7107	0.0020	− 0.4199
4	0.3445	0.7496	− 1.0078	0.1604	0.1913	0.6463	0.1051	0.7163	− 0.1292	0.7107	0.14518	− 0.4630
5	0.4806	0.7496	− 1.4575	0.1604	0.1550	0.6463	0.0915	0.7163	− 0.1038	0.7107	0.1231	− 0.4951
6	0.3183	0.7496	− 1.1558	0.1604	0.1679	0.6463	0.0815	0.7163	0.0428	0.7107	0.2348	− 0.5197
7	0.2984	0.7496	− 0.8245	0.1604	0.1599	0.6463	0.3073	0.7163	0.1162	0.7107	0.1548	− 0.5388
8	0.3921	0.7496	− 0.9588	0.1604	0.1601	0.6463	0.2947	0.7163	0.1785	0.7107	0.1082	− 0.5537
9	0.3865	0.7496	− 0.7381	0.1604	− 0.0754	0.6463	0.2692	0.7163	0.1542	0.7107	0.1683	− 0.5656
10	0.3962	0.7496	− 0.5348	0.1604	− 0.0582	0.6463	0.2538	0.7163	0.2122	0.7107	0.0581	− 0.5750
11	0.4983	0.7496	− 0.7928	0.1604	− 0.0476	0.6463	0.1805	0.7163	0.3364	0.7107	− 0.0586	− 0.5825
12	0.4923	0.7496	− 0.6008	0.1604	− 0.0377	0.6463	0.2018	0.7163	0.3762	0.7107	− 0.0023	− 0.5885
13	0.4952	0.7496	− 0.4669	0.1604	− 0.0443	0.6463	0.2256	0.7163	0.2221	0.7107	− 0.0118	− 0.5933
14	0.4393	0.7496	− 0.4519	0.1604	− 0.0333	0.6463	0.2950	0.7163	0.2844	0.7107	− 0.3125	− 0.5972
15	0.3718	0.7496	− 0.4373	0.1604	− 0.0360	0.6463	0.2815	0.7163	0.2693	0.7107	− 0.2255	− 0.6003
16	0.4004	0.7496	− 0.4336	0.1604	− 0.0259	0.6463	0.4308	0.7163	0.3727	0.7107	− 0.1214	− 0.6029
17	0.4180	0.7496	− 0.4159	0.1604	− 0.0295	0.6463	0.5571	0.7163	0.5505	0.7107	− 0.2366	− 0.6049
18	0.4228	0.7496	− 0.3892	0.1604	− 0.0341	0.6463	0.4555	0.7163	0.6221	0.7107	− 0.1738	− 0.6065
19	0.4465	0.7496	− 0.1972	0.1604	− 0.0341	0.6463	0.5158	0.7163	0.5834	0.7107	− 0.4366	− 0.6079
20	0.5964	0.7496	− 0.1327	0.1604	− 0.0376	0.6463	0.7272	0.7163	0.7070	0.7107	− 0.3381	− 0.6089
21	0.6592	0.7496	− 0.0346	0.1604	− 0.0268	0.6463	0.7262	0.7163	0.7077	0.7107	− 0.4765	− 0.6098
22	0.7068	0.7496	0.0835	0.1604	0.1684	0.6463	0.6266	0.7163	**0.7784**	0.7107	− 0.4154	− 0.6105
23	0.6331	0.7496	0.0468	0.1604	0.3442	0.6463	0.5826	0.7163	**0.7421**	0.7107	− 0.4703	− 0.6111
24	0.6892	0.7496	0.1374	0.1604	0.5025	0.6463	**0.8957**	0.7163	0.6526	0.7107	− 0.4451	− 0.6115
25	0.6789	0.7496	0.0732	0.1604	**0.7873**	0.6463	**0.8310**	0.7163	0.6032	0.7107	− 0.4461	− 0.6119
26	0.7192	0.7496	0.1460	0.1604	**1.0151**	0.6463	**0.7608**	0.7163	0.4975	0.7107	− 0.5180	− 0.6122
27	**0.9053**	0.7496	**0.1830**	0.1604	**1.1974**	0.6463	**0.7674**	0.7163	0.3718	0.7107	− 0.6078	− 0.6124
28	**0.7756**	0.7496	**0.26109**	0.1604	**1.3797**	0.6463	**0.7558**	0.7163	0.3954	0.7107	**− 0.6643**	− 0.6126
29	**0.7004**	0.7496	**0.3446**	0.1604	**1.5164**	0.6463	**1.0618**	0.7163	0.4415	0.7107	− 0.5034	− 0.6128
30	**0.5779**	0.7496	**0.3696**	0.1604	**1.6190**	0.6463	**0.9712**	0.7163	0.3703	0.7107	− 0.3541	− 0.6129

*Bold values indicate the OOC process.

Figure 1 presented the EWMCV-RSS chart indicating the process is OCC at 28^th^ observation whereas Fig. 2 presented the AEWMCV-SRS control chart indicating that the process is OCC at 27th. Figure 3 presents AEWMCV-RSS control chart and indicates the process is OCC at 27^th^ observation, and the process is indicated as OCC at 25th observation with AEWMCV-ERSS control in Fig. 4. Figure 5 presents the AEWMCV-MRSS control chart indicating the process is OCC at the 24th observation. Figure 6 presented the AEWMCV-QRSS control chart is OCC at 22nd observation. From Figs. 2, 3, 4, 5 and 6, it is revealed that the AEWMCV control chart detected the OOC signals at the 17th observation. From these results, it is inferred that the proposed AEWMCV control charts with suggested schemes have better performance than the EWMCV-RSS control chart.

## Conclusion

The AEWMCV-RSS chart has proven to be very useful and appropriate in situations where the ranking of items or units cannot be disregarded or where there aren't enough units available for sampling. It is therefore a sensible option for many real-world applications. We found that, particularly under RSS schemes, the performance of the suggested control charts (SRS and RSS-based AEWMCV) outperformed that of the EWMA-based CV control chart in terms of ARLs and SDRLs. This was based on a comparative analysis and a real-world scenario. This suggests that for all sorts of process shifts, the suggested control charts are more capable of identifying out-of-control (OOC) signals. In conclusion, the study's findings show that the SRS and RSS-based AEWMCV control charts outperform the conventional EWMA-based CV control chart in terms of performance. These proposed control charts can help with process monitoring and variability detection, which makes them useful tools for attempts to enhance quality across a range of industries and applications.

## Data Availability

The datasets used and/or analyzed during the current study are available from the corresponding author upon reasonable request. Further, no experiments on humans and/or the use of human tissue samples were involved in this study.
